# *Lactiplantibacillus paraplantarum* BPF2 and *Pediococcus acidilactici* ST6, Two Bacteriocinogenic Isolated Strains from Andalusian Spontaneous Fermented Sausages

**DOI:** 10.3390/foods12132445

**Published:** 2023-06-21

**Authors:** José David García-López, Claudia Teso-Pérez, Antonio Manuel Martín-Platero, Juan Manuel Peralta-Sánchez, Juristo Fonollá-Joya, Manuel Martínez-Bueno, Alberto Baños

**Affiliations:** 1 Departamento de Microbiología, Facultad de Ciencias, Universidad de Granada, Campus de Fuentenueva s/n, 18071 Granada, Spain; jdgarcia@correo.ugr.es (J.D.G.-L.); claudiatp@ugr.es (C.T.-P.); ammartin@ugr.es (A.M.M.-P.); jmps@ugr.es (J.M.P.-S.); abarjona40@gmail.com (A.B.); 2 Departamento de Zoología, Facultad de Biología, Universidad de Sevilla, Avenida de la Reina Mercedes 6, 41012 Seville, Spain; 3 Departamento de Nutrición y Bromatología, Facultad de Farmacia, Universidad de Granada, Campus Universitario de Cartuja s/n, 18071 Granada, Spain; 4 Instituto de Biotecnología, Universidad de Granada, 18071 Granada, Spain

**Keywords:** lactic-acid bacteria, fermented meat products, Leucocin K, pediocin, bacteriocin

## Abstract

Traditional spontaneously fermented foods are well known for their sensory and safety properties, which is mainly due to their indigenous microflora. Within this group of food, Mediterranean dry-cured sausages stand out as a significant source of lactic-acid bacterial strains (LAB) with biotechnological properties, such as their antimicrobial activity. The aim of this study was to investigate the biodiversity of antagonistic LAB strains from different Andalusian traditional sausages, such as *salchichón* and *chorizo*. First, a screening was carried out focusing on the antimicrobial activity against foodborne pathogens, such as *Listeria monocytogenes*, *Escherichia coli*, *Clostridium perfringens,* and *Staphylococcus aureus*, selecting two strains due to their higher antibiosis properties, both in agar and liquid media. These bacteria were identified as *Lactiplantibacillus paraplantarum* BPF2 and *Pediococcus acidilactici* ST6. In addition, genomic studies confirmed the presence of certain structural genes related to the production of bacteriocins. Finally, the culture supernatants of both strains were purified and analyzed by LC-MS/MS, obtaining the relative molecular mass and the amino acid sequence and identifying the peptides as the bacteriocins Pediocin-PA and Leucocin K. In conclusion, genomes and antimicrobial substances of *P. acidilactici* ST6, a Pediocin-PA producer, and *Lpb. paraplantarum* BPF2, a Leucocin K producer, isolated from Andalusian *salchichón* and *chorizo*, respectively, are presented in this work. Although further studies are required, these strains could be used alone or in combination as starters or protective cultures for the food industry.

## 1. Introduction

Fermentation is one of the oldest known preservation methods and has been one of the most used in Mediterranean countries since ancient times [1,2,3]. Spontaneously fermented meat sausages have a long tradition in Mediterranean countries as one of the most important traditional foods consumed throughout Europe [4]. Spanish fermented meat sausages such as *chorizo* or *salchichón* are generally made with small pieces of pork, back fat, sodium chloride, sodium nitrate-nitrite, and different spices, which are mixed homogeneously and stuffed into casings. The fermentation/maturation process occurs by storing the sausage in a well-ventilated chamber for 20–30 days under approximate temperature conditions of 25 ± 2 °C and 75–80% relative humidity. These conditions stimulate acidification and dehydration. The participation of different groups of bacteria is required for the start of the fermentation, including homofermentative lactic-acid bacteria (LAB). These bacteria are responsible for the typical characteristics of fermented meat products, such as flavor and color, as well as contributing to the formation of different metabolites such as organic acids, peroxides, aldehydes, etc. [5].

The production of many traditional food products obtained by spontaneous fermentation, particularly in the case of sausages, involves unspecified microbiota present in the raw materials or in the manufacturing process. Currently, sausages are produced in the industry with the use of a mixture of different starter cultures to provide a standardized flavor as well as microbiological and toxicological safety. These starter cultures are identified microorganisms, previously characterized as safe and exhibiting the desired metabolic activity [6]. In recent studies, the most promising microorganisms used as starter cultures are those isolated from the native microbiota of artisanal/local products. The reason for this is that they adapt well to ecological, environmental, and processing conditions, so they can develop more efficiently and dominate the microbiota present in the products [7,8]. In addition, the use of specific fermentations has different advantages: the enzymatic profile of these microorganisms can contribute to the production of sausages with the typical characteristics of the region [9,10]. They are capable of developing in a wide range of temperatures and tolerating adverse conditions, including the presence of sodium chloride, sodium nitrite, and acidic pH [11]. Nevertheless, apart from their impact on the fermentation process, it is crucial to examine the biopreservation abilities of starter cultures that produce bacteriocins to combat spoilage microorganisms and pathogens. [2,12,13].

Bacteriocins, especially those produced by lactic-acid bacteria (LAB), have attracted the most attention as tools for food biopreservation. Bacteriocins are antimicrobial peptides or proteins synthesized on ribosomes [14] that are not toxic to eukaryotic cells and are generally recognized as safe substances (GRAS). LABs are well known for their ability to produce a wide variety of bacteriocins [15] with antimicrobial activity against several pathogens such as *Listeria monocytogenes* or *Clostridium perfringens*, among others [16]. There is a growing consumer concern regarding the use of natural preservatives to enhance food safety. In light of this, bacteriocins hold great potential as a promising alternative to replace or reduce the dependence on chemical additives [17,18,19].

In recent years, traditional manufactured fermented foods such as cured sausages, cheeses [20], nham [12], or sucuk [21] from all over the world have been examined for bacteriocin-producing LABs with bioprotective application in food. Due to the limited knowledge of spontaneous fermentation processes in traditional meat sausages from the Mediterranean region, it would be suitable to further investigate these foods as natural sources of innovative strains with biotechnological applications. In the Andalusian region, there is a wide variety of spontaneously fermented sausages that may contain this kind of bacteria. In addition, these wild strains are anticipated to demonstrate enhanced adaptation to the specific attributes of these meat products and could compensate for the impoverishment of organoleptic characteristics due to the addition of commercial starter cultures [22].

Accordingly, the aim of this work was to study the potential antagonistic bacteria present in different spontaneously fermented sausages from Andalusia, focusing on the antimicrobial properties of LAB and selecting those strains that could contribute to improving safety against pathogenic bacteria, enabling their potential use as starter or bioprotective cultures.

## 2. Materials and Methods

### 2.1. Indicator Bacterial Strains and Culture Conditions

Indicator bacteria were obtained from the Spanish Collection of Type Cultures (CECT) (Valencia, Spain) and the German Collection of Microorganisms and Cell Cultures (DSMZ) (Braunschweig, Germany), which are listed in Table 1. Each bacterium was grown on a specific culture medium, indicated by the culture collection to which each one belongs. All strains were cultivated routinely on either trypticase in soy broth (TSB) (Scharlau, Barcelona, Spain) at 37 °C, and stored at 4 °C on the respective agar slants.

### 2.2. Isolation of LAB Species from Dry-Fermented Sausages

LAB strains were isolated from traditional dry sausages collected from Andalusia, in the south of Spain. The isolation of LAB was performed based on the method described by Zhu [13]. A total of 14 samples of artisanal *salchichón* and *chorizo* from different locations of Andalusia (Spain), produced without starter addition, were collected: a *salchichón* from Alhendín (SA), a *salchichón* from Bérchules (SB), a *salchichón* from Écija (SE), a *salchichón* from Olvera (SCT), a *salchichón* from Grazalema (ST), a *salchichón* from Baños de la Encina (SBE), a *chorizo* from Bérchules (CPN), a *chorizo* from Chirivel (CCH), a *chorizo* from Prado Negro (BPF), a *chorizo* from Ubrique (CCU), a *chorizo* from Cazorla (CCA), a *chorizo* from Écija (CHE), a *chorizo* from Órgiva (CD), and a *chorizo* from Olvera (CT). For sampling purposes, we collected three samples from each sausage: two samples from the distal ends and one sample from the central portion. Sausage samples (25 g) were cut into pieces and added to 225 mL of sterile saline solution (0.85%, *w*/*v*). The samples were processed in sterile bags with filters (GASPAK Cromakit, Granada, Spain), and homogenized for 3 min in a paddle homogenizer (MASTICATOR, IUL Instruments, Barcelona, Spain). Subsequently, 0.1 mL of each 10-fold dilution series was spread on Man Rogosa Sharpe Agar, medium for selective isolation and culture lactic-acid bacteria (MRS Agar, Scharlab, Barcelona, Spain) and incubated at 30 °C for 48 h. Colonies were selected by streak plating three times until uniform colonies were obtained as candidate LAB strains. Candidate isolates were stored at refrigeration temperature before use.

### 2.3. Screening and Selection of Antagonist LAB Strains

Antimicrobial activity by candidate LAB strains was determined by the diffusion agar method according to Alonso et al. [23], against *L. monocytogenes* DSM 112142, *S. aureus* CECT 520, and *E. coli* CECT 516. Bacteria were grown in the general enriched media Brain Heart Infusion (BHI, Scharlab, Barcelona, Spain). The pathogenic cultures were incubated overnight at 37 °C. Then, these bacteria were spread in Brain Heart Infusion Agar (BHA, Scharlab) dishes using a bacterial suspension adjusted to 1 × 10^6^ CFU/mL to form a bacterial lawn. Once the plate was dried, drops of 10 µL of different isolated strains. After incubation at 30 °C for 48 h, the plates were examined to determine the absence/presence of inhibition zones and results were interpreted as positive (+) or negative (−). For positives, three levels of intensity were established depending on the diameter of the inhibition zone: + (1–10 mm); ++ (11–15 mm); +++ (>15 mm). Independent tests were carried out in duplicate for each pathogenic bacterium. From this point forward, a screening process was initiated to select the isolates that demonstrated higher antibacterial activity.

The next step was to detect the production of extracellular antimicrobial inhibitors by each of the isolates that previously produced antibiosis in a solid medium against the target pathogens. Thus, the selected strains were inoculated in sterile tubes with 9 mL of BHI and incubated for 24 h at 30 °C. Then, 1.5 mL of each culture was centrifuged (13,000× *g*) and filtered using 3 mL syringes to which was attached a polyethersulfone (PES) filtration membrane (Merck Millipore, Carrigtwohill, Ireland) with a pore size of 0.22 μm, the filtrates were collected in sterile microcentrifuge tubes. To assess the antimicrobial activity of the filtrates, the well technique described by Tagg and McGiven [23,24] was employed, using *L. monocytogenes* CECT 4032, *Cl. perfringens* CECT 821, and *S. aureus* CECT 239 as the target bacteria [24,25]. All target bacteria were incubated at 30 °C for 24–48 h. *Cl. perfringens* was incubated under anaerobic conditions (AnaeroGen; Thermo Fisher Scientific, Landsmeer, The Netherlands). Each sample was tested in duplicate. Once the indicator strain has grown, the appearance of inhibition halos around the wells was observed and measured in millimeters. Therefore, we were able to determine if the strain had produced the compound responsible for the antimicrobial activity observed in culture supernatants. Finally, the two strains that exhibited greater antibiosis and a broader spectrum of inhibition against target strains were selected.

### 2.4. Strain Identification and Characterization

Identification was based upon phenotypic characteristics, including cell morphology and Gram-staining, catalase activity, API50 system (BioMérieux, Craponne, France), ability to grow at 10 and 45 °C and the presence of 6.5% (*w*/*v*) NaCl.

For genotypic characterization, genomic DNA was extracted from pure cultures according to Martín-Platero et al. [26]. Identification of the selected strains was carried out by searching homologies of 16S ribosomal RNA in the BLASTN database (National Center for Biotechnology Information) using BLAST [27]. In addition, both genomes were sequenced with the Illumina HiSeq4000 platform by STAB VIDA (Caparica, Portugal), assembled with SPAdes 3.13 [28], and annotated with Prokka 1.13.3 [29]. Homologies between gene clusters were revealed by aligning in pairs using Blastn suit-2 sequences [30]. Functional analysis of genomes was performed through InterProScan v5.50-84.0 [31,32], which classifies proteins into families, predicting domains and important sites. InterproScan generates Gene ontology terms associated with each gene. Afterwards, the Gene Ontology Database [33,34,35] was used to perform a functional analysis of genes and their products.

Finally, to determine if the genomes were associated with the structural gene of any bacteriocin, a tblastn (version 2.10.1+) was run with a 10–6 e-value threshold [36,37] between our genomes and bacteriocins from the BACTIBASE databases [38].

### 2.5. Bacteriocin Production and Purification Assay

First, production in a liquid medium and purification by cation exchange chromatography was performed. Therefore, flasks with 1 L of MRS medium in 0.1 mol/L sodium phosphate buffer (pH 7.2) were inoculated at 5% with an overnight culture of each of the strains of interest (culture in stationary phase). The cultured flasks (1 L) were incubated at 30 °C overnight and centrifuged for 20 min at 4 °C and 4750 rpm, collecting the supernatant to assess their inhibition by the well technique [24]. The recovery of the bacteriocins was carried out following Abriouel et al. [39]. The supernatants at pH7 were mixed with 1 N NaOH, 200 mL of Carboxymethyl Sephadex CM-25 (GE Healthcare, Madrid, Spain), and stirred for 30 min. Then, they were left to settle for 30 min. Afterwards, the supernatants were removed, and the CM-25 gel was transferred to a cylindrical filtering funnel with a plate with a porosity of 100–160 microns (Pobel, Madrid, Spain). The gel was washed with three volumes of distilled water, followed by two volumes of 1 M NaCl and two volumes of 1.5 M to elute the adsorbed bacteriocin. During the process, 50 mL fractions were collected manually, these were sterilized by filtration 0.22 μm PES (Merck Millipore, Cork, Ireland), and the activity was measured using the abovementioned well technique.

Subsequently, the determination of the molecular weight of both bacteriocins was carried out by LC-MS/MS. For this purpose, a tricine-sodium dodecyl sulfate-polyacrylamide gel electrophoresis (SDS-PAGE) (Sigma-Aldrich, Madrid, Spain) system was used [40]. A total of 20 µL of the sample was mixed with 6 µL of Laemmli sample buffer (Bio-Rad), heated to 100 °C for 3 min and cooled to room temperature. The mixtures were injected into the wells of the 12% precast polyacrylamide gel (Criterion TGX™, Bio-Rad, Hercules, CA, USA), using standard proteins as molecular weight markers (Spectra™ Multicolor Low Range Protein Ladder, Thermo Scientific™, Madrid, Spain), Electrophoresis was carried out using the Criterion™ Cell (300 V, 20 min) (Hercules, CA, USA). The gel was washed with sterile distilled H_2_O and divided into three equal-sized fragments, each including a band of molecular weight markers. Two of the gel fragments were fixed with 25% (*v*/*v*) isopropanol and 10% (*v*/*v*) glacial acetic acid (Sigma) for 4 h, then washed with sterile distilled H_2_O. One of the fixed gels was stained with Coomassie blue (Sigma) overnight with constant shaking at room temperature. Finally, a solution of water and methanol was used to attenuate the gel. The bands were revealed by a gel documentation system. For the antimicrobial activity test, the gel fragment without fixation process was placed in a sterile Petri dish, then covered with BHA agar containing the indicator strain *L. monocytogenes* DSM 112142. The dish was kept at 4 °C for 30 min and then incubated at 30 °C overnight. The last sample of fixed gel was stored in sterile distilled H_2_O for identification by LC-MS/MS.

Molecular mass determination and amino acid analysis of the bacteriocins from two selected strains were carried out by the Proteomics Unit at the López-Neyra Institute of Parasitology and Biomedicine, (IPBLN-CSIC, Granada, Spain). The pieces of gel that showed antimicrobial activity and contained the proteins of interest, were analyzed by LC-MS/MS. For this, they were cut manually and digested with trypsin gel (Promega, Madison, WI, USA) using a Digest MSPro (Intavis, Koeln, Germany) following standard procedures. Briefly, gel slices were reduced with 10 mM DTT, alkylated with 55 mM iodoacetamide, dehydrated with acetonitrile, and then digested with trypsin for 18 h at 30 °C. Peptides were extracted with 0.2% TFA and eluted with 30% acetonitrile (Sigma). The eluates from both extractions were dried in a vacuum centrifuge and stored at −20 °C. Analysis by mass spectrometry (LC-MS/MS) was performed by nLC (easy nanoLC, Proxeon, Thermo Fisher) coupled to an ion trap type mass spectrometer (Amazon Speed ETD, Bruker, Madrid, Spain) equipped with a captive source. The chromatographic separation was carried out on a C18 column (15 μm × 15 cm, 3 μm, 100 A) using a flow 300 nL/min with gradients from 5 to 30% B in 120 min (buffer A: 0.1% Fluoroacetic (FA) in water; buffer B: 0.1% FA in AcN). The mass analysis has been carried out in the range of 390–1400 (*m*/*z*). A total of 10 precursors per cycle were selected for fragmentation, establishing a dynamic exclusion of 0.5 min. Protein identification was performed using the ProteinScape program (Bruker) and MASCOT (Matrix Science, Boston, MA, USA) as search engines. The search was carried out using the Swiss-Prot database with a filter for the bacteria isolates, both downloaded from UniProt. In all cases, carbamidomethylation was considered to be a fixed modification, and oxidation was a variable modification.

### 2.6. Bacteriocin Stability against Heat, pH, and Enzymes

The two bacteriocin-producing strains were grown in 0.5 L of MRS broth (30 °C, 24 h) then 4750 g centrifuged at 4 °C for 35 min and filtered through a 500 mL Vacuum Filtration System, (0.22 μm PES Membrane, VWR International) to obtain a cell-free filtrate. The methodology indicated by Ahn et al. [41] was followed to test pH and thermal stability, the cell-free filter was adjusted to pH 6.5 with 4 N-HCl or NaOH and then heated at 60 °C, 80 °C, and 100 °C for 30 min each, or autoclaved (121 °C, 15 min). The activity was examined at various pHs by adjusting the cell-free filtered to pH 2, 3, 5, 7, 9, and 11 with 4 N-HCl or NaOH, which was then held at 30 °C for 2 h. Each sample was neutralized to pH 6.5. The samples were then membrane filtered (PES filter of 0.22 μm) and analyzed by the agar well diffusion assay as described above. Enzymatic degradation assay as proposed by Zhao et al. [42] was carried out to verify the protein nature of the inhibitor produced by the bacteria under study. For this, three proteinase enzymes were used: papain (≥10 units/mg, Sigma), trypsin (2000 units/mg, Sigma), and proteinase K (30 units/mg, Sigma). For each bacteria cell-free filter, the enzymes were added to each one at a concentration of 0.5 mg/mL and incubated at 37 °C for 2 h [43,44]. They were heated at 80 °C for 5 min to make those enzymes inactivated. After that, the antimicrobial activity was tested against *L. monocytogenes* DSM 112142 using the previously described well diffusion agar technique.

### 2.7. Antagonism Assays in Cocultures

The inhibition of foodborne pathogen growth was evaluated following Baños et al. [45]. Overnight cultures of the two LAB-selected strains and pathogenic strains (*L. monocytogenes* CECT 4032, DSM 112142, and *C. perfringens* CECT 821) were diluted at 10^3^ CFU/mL into BHI (Scharlab), incubated at 30 °C and shaken at 130 rpm, individually and in cocultures [46]. In the case of *Cl. perfringens*, the samples were incubated under anaerobic conditions (AnaeroGen; Thermo Fisher Scientific). At selected times of 0, 1, 5, and 10 days, samples were collected and serially diluted into a sterile saline solution. Dilutions were plated in triplicate into MRS Agar for LAB bacteria, Compass^®^ Listeria Agar (Biokar Diagnostics SA, Allonne, France) and CHROMagar^®^ C. perfringens (Scharlab), two chromogenic and selective media for quantification of *L. monocytogenes* and *C*. *perfringens*, respectively. The average number of colonies obtained after 48 h incubation at 30 °C was used to establish the growth curves of those bacteria cultured alone and co-cultivated.

### 2.8. Statistical Analysis

Statistical analysis and figures were performed with GraphPad Prism 8.0 software (GraphPad Software Inc., San Diego, CA, USA). All results were expressed as the mean ± standard deviation (SD). Differences between means were tested for statistical significance using a one-way analysis of variance (ANOVA).

## 3. Results and Discussion

### 3.1. Isolation of LAB Species from Dry-Fermented Sausages

The microbiota present in 14 different samples of sausages from Andalusia was explored focusing on lactic-acid bacteria (LAB) with potential biopreservation properties [3,17,47,48]. A total of 640 colonies were obtained on the MRS agar, which were considered potential candidates for antagonistic LAB.

### 3.2. Screening and Selection of Antagonist LAB Strains

The antimicrobial activity of the 640 initially isolated strains was evaluated using the agar diffusion method, obtaining a total of 62 strains that produced some inhibition against target bacteria *L. monocytogenes*, *S. aureus,* or *E. coli* (Table 2). As expected, the tested strains showed remarkable activity against Gram-positive bacteria [47,49,50] but did not exhibit activity against Gram-negative bacteria. In a second screening, 12 bacteria (Table 2) that showed the highest antagonist activity against the target bacteria were selected. These bacteria were employed to perform extracellular antimicrobial activity assays according to the previously described methodology, and the findings are reported in Table 3.

All strains exhibited antibiosis against the three target strains, with most of them being particularly active against *L. monocytogenes* (Table 3). The BPF2 and ST6 strains were ultimately chosen due to their significant anti-Listeria activity as well as their inhibitory effects against *S. aureus* and *Cl. perfringens*.

After selecting the two strains with the most favorable antimicrobial properties, an antibiosis test was performed, confirming the absence of antagonism between both strains and therefore allowing for their potential combined use as starters cultures [51,52].

### 3.3. Identification and Genomic Studies

Before carrying out the genomic studies, the phenotypic characterization revealed that ST6 and BPF2 strains were Gram-positive cocci and bacilli, respectively. In addition, initial identification using the API50 system indicated that ST6 was associated with *Pediococcus* spp., while BPF2 was related to the *Lactobacillus* genus. Both strains were catalase-negative and demonstrated the ability to grow within a temperature range of 20–40 °C and in the presence of 6.5% (*w*/*v*) NaCl.

After sequencing, assembly, and annotation, analysis of the 16S gene predicted that our strains ST6 and BPF2 correspond to *Pedicoccus acidilactici* and *Lactiplantibacillus paraplantarum* species, respectively. The genome of *P. acidilactici* has a size of 1.95 Mb, with a GC content of 42.18%, while *Lpb. paraplantarum* genome has a size of 3.48 Mb with a GC content of 43.6%. These values are similar and within the normal range reported in the literature for most of the *P. acidilactici* and *Lpb. paraplantarum* strains [53,54,55,56,57]. Other characteristics of these genomes are summarized in Table 4.

**Figure 1:**
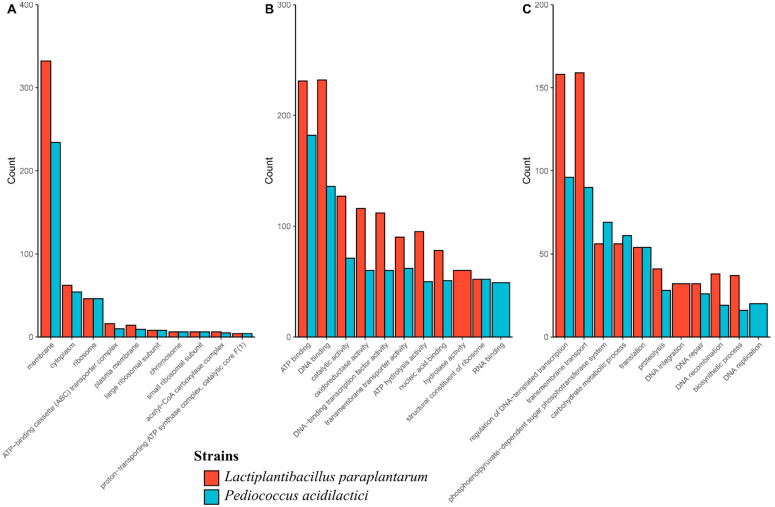
Ten most abundant GO terms in (**A**) cellular component, (**B**) molecular function, and (**C**) biological processes categories.

Gene Ontology (GO) analysis showed that the cellular component category membrane was the most abundant term in both genomes (Figure 1A). In the molecular function category, DNA binding and ATP binding were the most abundant terms in *Lpb. paraplantarum* and *P. acidilactici*, respectively (Figure 1B). In addition, in the biological processes category, the regulation of DNA-templated transcription prevailed in *P. acidilactici*, while transmembrane transport was in *Lpb. paraplantarum* (Figure 1C). Other GO results are summarized in Table 5.

**Figure 2:**
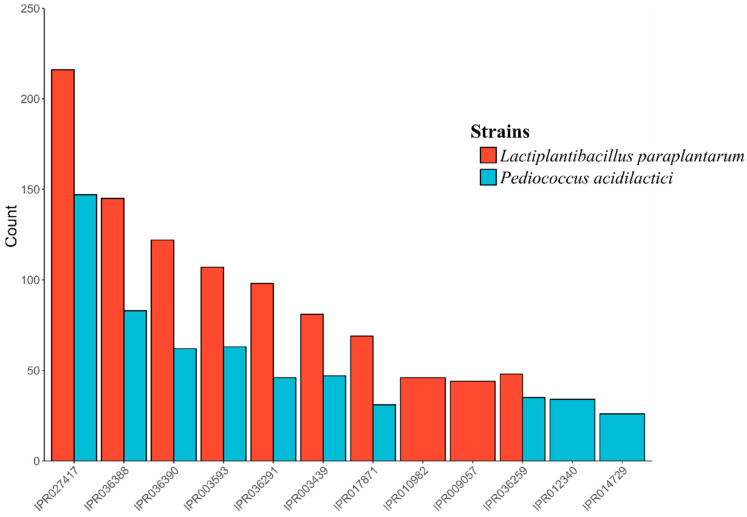
Top ten most abundant InterProScan gene families for: (red) *Lactiplantibacillus paraplantarum* BPF2 genome; (blue) *Pediococcus acidilactici* ST6 genome.

InterProScan classified 1721 and 2850 proteins from *P. acidilactici* and *Lpb. Paraplantarum*, respectively. A total of 6059 families were assigned to 1671 of the total proteins (97.1%, 1671/1721) in *P. acidilactici*. In the case of *Lpb. paraplantarum*, 9049 families were assigned to 2716 (95.3%, 2716/2850) of the total proteins. In all cases, the most abundant domain was the P-loop-containing nucleoside triphosphate hydrolase family (IPR027417), followed by the winged helix-like DNA-binding domain superfamily (IPR036388 and IPR036390) and the AAA+ ATPase domain (IPR003593) (Figure 2).

**Figure 3:**
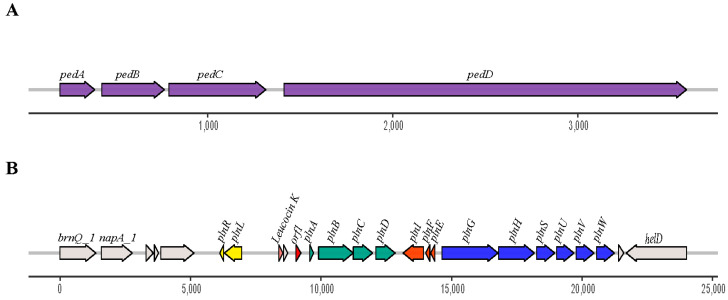
Genetic organization of (**A**) Pediocin-PA gene cluster found in both strains and (**B**) *pln* loci found in *Lpb. paraplantarum* BPF2. The Pediocin-PA cluster is formed by four genes, *pedABCD*. In the case of the *pln* loci, it is formed by at least 15 genes of which *plnA* codes for the Plantaricin A and *plnEF* for the plantaricins E and F, respectively. Additionally, the gene that encodes Leucocin K bacteriocin is present in the genome of *Lpb. paraplantarum* BPF2.

The results of tblastn against the BACTIBASE database showed that Pediocin-PA was found in both genomes. In addition, five other bacteriocins were found in the *Lpb. paraplantarum* BPF2 genome: Mutacin III/1140, Leucocin K, Plantaricin A, Plantaricin E, and Plantaricin F. Furthermore, the gene clusters responsible for the production of bacteriocin were found (Figure 3A,B).

**Figure 4:**
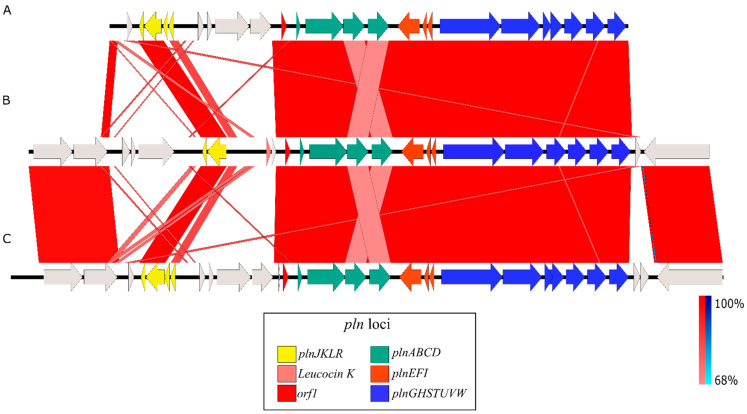
Homology between *pln* loci from (**A**) *L. plantarum* C11, (**B**) *Lpb. paraplantarum* BPF2, and (**C**) *L. plantarum* WCF1. The color scale represents the similarity between the different genes: light red and blue = 68% similarity; dark red and blue = up to 100% similarity; red corresponds to direct similarity and blue to inverse similarity. Similarities found upstream and downstream between *Lpb. paraplantarum* BPF2 and WCF1 correspond, respectively, to the genes *brnQ1* and *napA1*, which are not part of the *pln* regulon, and to the *helD* gene that encodes for a DNA helicase IV.

The Pediocin-PA gene cluster was formed by four genes, *pedABCD*, in both cases. The Plantaricin A gene cluster found in *Lpb. paraplantarum* BPF2 shows high similarities to the *pln* loci previously described from *Lactobacillus plantarum* C11 and WCFS1 [58,59] (Figure 4). The *pln* locus contains five inducible operons: *plnABCD*, *plnEFI*, *plnJKLR*, *plnMNOP*, and *plnGHSTUVW*. In our *Lpb. paraplantarum*, *plnABCD*, *plnEFI*, and *plnGHSTUVW* operons are present, but the list is not complete. Operon *plnJKLR* appears to be truncated as it lacks the bacteriocin genes *plnJK*. *plnT* gene and *plnMNOP* operons are absent. The *plnEFI* genes code for two-peptide bacteriocin (plantaricins EF) and their cognate immunity protein (*PlnI*). *plnLR* encodes for a putative immunity protein containing a protease CAAX signature and a putative protein with an unknown function, respectively. *plnGH* codes for an ABC-transporter and an accessory protein, respectively. Both of these constitute an ABC-transport system involved in peptide secretion, utilizing a double-glycine leader. *plnSTUVW* exhibits considerable homology among itself and with proteins belonging to the type II CAAX amino protease family. Finally, *plnABCD* codes for a quorum-sensing network that is essential for expressing all the genes in the *pln* locus [60], where the pheromone peptide, Plantaricin A, is involved. In addition, the gene responsible for Leucocin K appears upstream of *plnABCD* in *Lpb. paraplantarum* (Figure 3B).

Numerous bacteriocinogenic strains of *P. acidilactici* have been isolated from fermented food products derived from both plants and animals. Furthermore, these strains have been extensively studied for their probiotic and biopreservative properties [17]. Consistent with our findings, some authors have also reported the isolation of *Lpb. paraplantarum* strains with biotechnological interest, including bacteriocin production, from spontaneously fermented foods such as fermented mushrooms, Chinese pickles, and Korean fermented foods [61,62,63].

### 3.4. Identification of the Bacteriocins by LC-MS/MS

**Figure 5:**
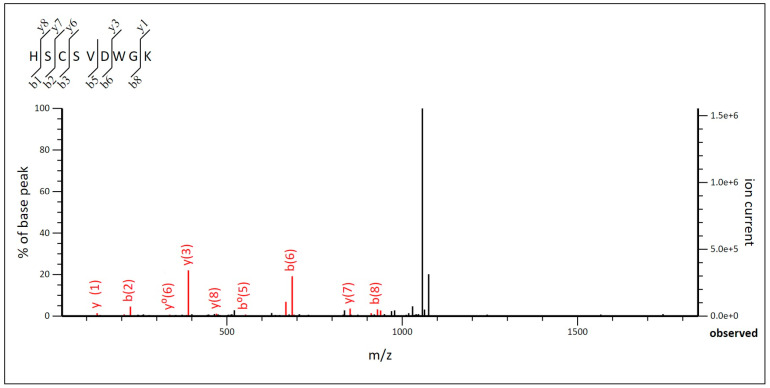
MS/MS diagram of bacteriocin of *Pediococcus acidilactici* ST6.

In this study, an SDS-PAGE was performed on the samples that showed the highest activity after the purification step. The SDS-PAGE gel coupled to an antimicrobial activity test showed a zone of inhibition in the lanes corresponding to the *P. acidilactici* ST6 and *Lpb. paraplantarum* BPF2 samples. The bands responsible for the zone of inhibition corresponded to a molecular weight in both cases between 10 and 4.6 kDa. The amino acid sequence and the molecular mass were analyzed using an LC-MS/MS system. The results obtained in the case of the bacteriocin from *P. acidilactici* ST6 were 5378 Da for the molecular mass. The MS/MS graph was analyzed (Figure 5) to determine the amino acid sequence of ST6 as a function of the ionic strength of each fragment. The amino acid sequence of bacteriocin ST6 was His-Ser-Cys-Ser-Val-Asp-Trp-Gly-Lys (HSCSVDWGK). Comparison with known proteins in the UniProt database revealed that the amino acid sequence of ST6 matches the amino acid sequence of the antimicrobial peptide Pediocin-PA. The results obtained are consistent with the genomic study carried out previously in which *P. acidilactici* ST6 presented only the Pediocin-PA gene, which belongs to class IIa, linear peptides with 25 to 58 residues containing one or two disulfide bridges [64]. These have been named the “family of pediocins” in honor of the first representative of this class and the most widely studied, pediocin-PA-1 [65].

**Figure 6:**
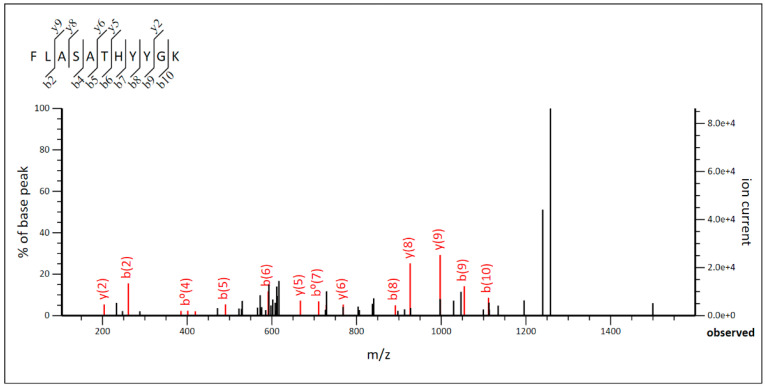
MS/MS diagram of bacteriocin of *Lactiplantibacillus paraplantarum* BPF2.

In the case of *Lpb. paraplantarum* BPF2, the molecular mass was 5825 Da, and the MS/MS graph (Figure 6) was analyzed to determine the amino acid sequence of the BPF2 peptide as a function of the ionic strength of each fragment. The amino acid sequence was Phe-Leu-Ala-Ser-Ala-Thr-His-Tyr-Try-Gly-Lys (FLASATHYYGK). Comparison with known proteins in the UniProt database revealed that the amino acid sequence matches the amino acid sequence of the antimicrobial peptide Leucocin K. The results obtained in the genomic study revealed genes to produce six different bacteriocins: Pediocin-PA, Mutacin III, Leucocin K, Plantaricin A, Plantaricin E, and Plantaricin F. However, the LC-MS/MS analysis revealed the presence of only Leucocin K, which presented a value of emPAI (exponentially modified protein abundance index) of 0.77 [66], which indicates a high concentration of Leucocin K in the analyzed sample. Finally, the amino acid sequence obtained by LC-MS/MS was compared with the encoded sequence in genomes of BPF2 and ST6 strains, confirming the identification of both bacteriocins.

The presence of Pediocin-PA genes in the genomes of *Lpb. paraplantarum* BPF2 and *P. acidilactici* ST6 could indicate the resistance of both strains to Pediocin-PA. On the other hand, although most of the Leucocin produced by *Leuconostoc* has been reported to exhibit anti-listerial activity, this fact is not extensible to other Gram-positive bacteria, which could explain the resistance of *P. acidilactici* ST6 to Leucocin K [67]. These aforementioned facts may indicate the compatibility of both bacteria and their potential synergistic use to produce fermented foods.

### 3.5. Effect of Temperature, pH, and Different Enzymes on Bacteriocins

The stability of the two bacteriocins was tested under different conditions of temperature, pH, and treatment with enzymes. The results presented in Table 6 show that Pediocin-PA activity was maintained in a wide pH range between 2 and 10 and it was sensitive to protease enzymes such as pepsin, papain, and trypsin. The antimicrobial activity was not affected by heating at 80 °C for 30 min. However, at higher temperatures, its activity began to reduce with a complete loss of efficacy observed at 121 °C for 15 min. This indicates that Pediocin is thermolabile [68,69,70]. In the case of Leucocin K from BPF2, results similar to those reported by other authors were obtained [71]. This bacteriocin was sensitive to the tested proteases and showed good thermal stability up to 80 °C similar to Pediocin, completely losing activity at 121 °C, with a wide active pH range from 2 to 10 without loss of efficacy (Table 6).

Other authors have recently reported bacteriocinogenic strains of *P. acidilactici* isolated from meat products. The strain *P. acidilactici* LMQS 154.1, isolated from traditionally produced fermented sausages in Germany, has been described [5]. Consistent with our findings, this strain also demonstrated significant anti-listerial activity associated with the production of Pediocin-PA-1 [5]. In another recent work, *P. acidilactici* ST3522BG isolated from silage has been identified as a pediocin-PA-1 producer with high antimicrobial activity against *Listeria* and vancomycin-resistant *Enterococcus* species [72]. Furthermore, *P. acidilactici* ST3522BG produces other antifungal metabolites with the potential for the inhibition of mycotoxigenic molds [72].

In relation to the bacteriocinogenic strains of *Lpb. paraplantarum*, recent studies have highlighted the capability of *Lpb. paraplantarum* RX-8, isolated from traditional pickles, to produce plantaricin, linked to its antibacterial activity [61]. Furthermore, other studies have highlighted the potential use of similar bacteriocinogenic strains of *Lactiplantibacillus plantarum* for animal nutrition, showing promising results in mitigating methane emissions in ruminants [73].

### 3.6. Antagonism Assays in Cocultures

**Figure 7:**
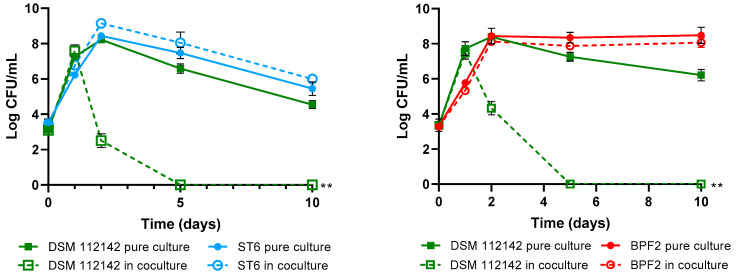

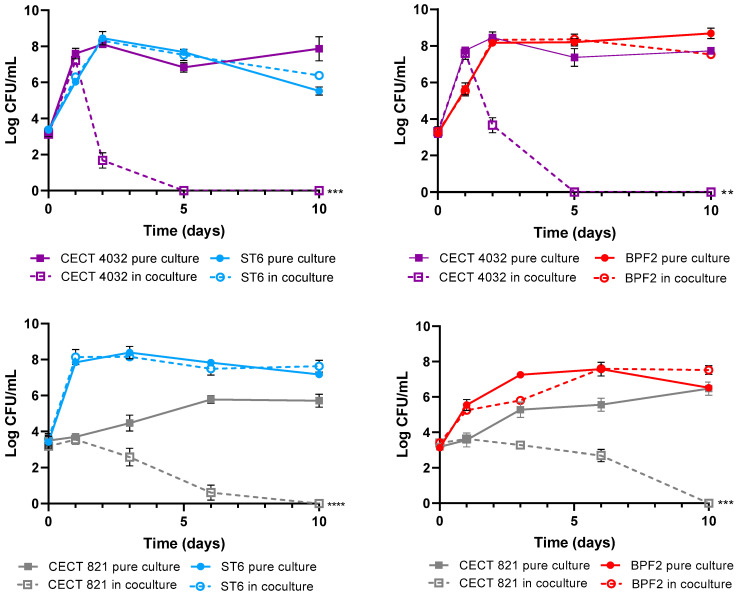
Bacterial survival in cocultures of *Pediococcus acidilactici* ST6 and *Lactiplantibacillus paraplantarum* BPF2 with *Listeria monocytogenes* DSM 112142 and CECT4032 and *Clostridium perfringens* CECT821. The bars represent means ± standard deviations of three independent tests. ** *p* < 0.01; *** *p* < 0.001; **** *p* < 0.0001 respect to controls.

The objective of these assays was to demonstrate the competitive exclusion of the *P. acidilactici* ST6 and *Lpb. paraplantarum* BPF2 against *L. monocytogenes* and *Cl. perfringens* pathogens. Competitive exclusion can be defined as the state in which coexisting bacterial species in the same ecological niche compete for limited resources such as nutrients and space through either competition or interference mechanisms [74]. In this instance, an interference mechanism was observed as both strains exhibited the ability to produce bacteriocins. As can be observed in Figure 7, the growth of LAB strains in both the control and the co-culture was similar, indicating that their growth is not affected by the presence of pathogens. Nevertheless, for *L. monocytogenes* DSM 112142 and CECT 4032, a rapid decline of bacterial counts was observed after 48 h, and complete absence was achieved after 5 days of coexistence with both lactic-acid bacteria strains. Although *Cl. perfringens* showed lower sensitivity compared to *Listeria*, a significant decrease in bacterial counts was observed during co-culture with both LAB strains, leading to the complete elimination of the pathogen after 10 days.

In accordance with our results, other authors have described the activity of pediocin-PA-producing strains for the control of *L. monocytogenes* in both in vitro and in efficacy trials in different food models [75,76,77,78,79]. Other studies have similarly described the ability of certain bacteriocinogenic strains of *Pediococcus* to inhibit *Clostridium* species [75,76,80]. On the other hand, Leucocins are bacteriocins specific to *Leuconostoc*, with very little evidence of their production in other LAB genera. This fact makes the *Lpb. paraplantarum* BPF2 strain particularly unique as Leucocin K producer. As with our results, Leucocin K7 from *L. mesenteroides* has demonstrated anti-Listeria activity in vitro and in foods such as milk [71].

## 4. Conclusions

A biodiversity study of spontaneously fermented Andalusian sausages revealed the significant potential of these foods as a source for the isolation of lactic-acid bacteria with interesting technological and antibiosis properties. This work conducted a screening study from hundreds of isolated strains obtained from artisanal *salchichones* and *chorizos*, selecting two strains for showing the broadest spectrum of antibiosis against food pathogens. *P. acidilactici* ST6, a pediocin-PA producer isolated from *salchichón*, and *Lpb. paraplantarum* BPF2, a Leucocin K producer isolated from *chorizo*, are presented in this work. Both strains exhibited significant inhibitory activity against *L. monocytogenes* and *Cl. perfringens*.

Further studies are required to delve into the genomic characteristics and their potential use in food models. In addition, it would be advisable to investigate their influence on the technological and sensory properties of foods in which they could be used as potential starters or protective cultures.

## Figures and Tables

**Table 1:** Indicator bacterial strains used for testing the antibiosis activity of potential probiotic strains.

Bacterial Strain	Reference	Isolation
*Listeria monocytogenes*	DSM 112142	Minced meat
*Listeria monocytogenes*	CECT 4032	Associated with a case of meningitis after eating soft cheese
*Staphylococcus aureus*	CECT 239	Human lesion
*Escherichia coli*	CECT 516	Human feces
*Clostridium perfringens*	CECT 821	Sheep

**Table 2:** Wild LAB isolates from Andalusian sausages, showing the results of antibiosis against target bacteria.

LAB Strain	*L. monocytogenes* DSM 112142	*S. aureus* CECT 520	LAB Strain	*L. monocytogenes* DSM 112142	*S. aureus* CECT 520
SE1	+	-	CPN1.1 *	+++	+
SE2	++	-	CPN1.3	+	+
SA01	++	+	CPN2.1	+	+
SE3.5	+	+	CPN2.2	++	-
SA4	++	-	CPN2.5	+	-
SA4.7	++	-	BPF1 *	++	++
SA4.7	++	+	BPF2 *	+++	++
SA5.2	+	-	SBE13.6	+	+
SA5.5	++	-	SBE 15.1	++	-
SA6.1	++	-	SBE 15.3	+	-
SA6.3	++	+	CT1.1	++	-
SA6.4	+	+	CT1.4	++	+
SB6.4	++	-	CT1.4	+	-
SB6.4	++	+	CT1.5	++	+
SB6.5	+	+	CCU1.1	+	-
SB7.3	+	+	CCU1.14	-	+
SB8.1	++	-	CCU2.2	+++	-
SB8.7	+	+	CCH2 *	+++	++
SB8.6	-	+	CCH4	+	+
SB9.7	++	+	CCH5	++	+
CHE1.2	++	+	CCH7	++	+
CHE 1.4	+	+	CCH9 *	+++	+
CHE 2.1	+	-	CCH11 *	+++	-
CHE 1.3	++	+	CT4.2	+	-
CCA1.2	-	+	ST5	++	+
CCA1.2 II	+	+	CD2	++	-
CCA 2.2	-	+	CD4	++	-
SCT7 *	+++	++	SCT1 *	+++	++
SCT9 *	+++	++	SCT3	+	-
ST2	++	-	ST7 *	+++	+
ST9 *	+++	+	ST6 *	+++	++

Diameter of inhibition zone; +, 1–10 mm; ++, 11–15 mm; +++, >15 mm, - absence. The initials indicate the type and origin of the spontaneously fermented sausage: (SA) *salchichón* from Alhendín, (SB) *salchichón* from Bérchules, (SE) *salchichón* from Écija, (SCT) *salchichón* from Olvera, (ST) *salchichón* from Grazalema, (SBE) *salchichón* from Baños de la Encina, (CPN) *chorizo* from Bérchules, (CCH) *chorizo* from Chirivel, (BPF) *chorizo* from Prado Negro, (CCU) *chorizo* from Ubrique, (CCA) *chorizo* from Cazorla, (CHE) *chorizo* from Écija, (CD) *chorizo* from Órgiva and (CT) *chorizo* from Olvera. The asterisk (*) indicates the isolates with the highest antimicrobial activity.

**Table 3:** Antimicrobial activity of the supernatant of the LAB strains against pathogenic bacteria. Results expressed as the average diameter ± standard deviation of the inhibition zone (mm).

LAB Strain	*L. monocytogenes* DSM 112142	*S. aureus* CECT 520	*C. perfringens* CECT 821
CPN1.1	15 ± 0.5	8 ± 0.5	10 ± 0.5
BPF1	16 ± 1	9 ± 1	11 ± 0.5
**BPF2**	**24 ± 0.5**	**16 ± 0.4**	**18 ± 0.5**
CCH2	18 ± 0.6	8 ± 0.5	12 ± 1
CCH9	19 ± 1	8 ± 1	10 ± 0.5
CCH11	16 ± 0.5	10 ± 0.5	12 ± 1
SCT7	19 ± 1	12 ± 1	11 ± 0.5
SCT9	20 ± 0.4	14 ± 0.5	13 ± 0.5
ST9	18 ± 1	6 ± 0.4	10 ± 1
SCT1	19 ± 0.5	9 ± 0.5	9 ± 1
ST7	20 ± 0.5	10 ± 0.4	12 ± 0.5
**ST6**	**24 ± 0.4**	**13 ± 0.4**	**15 ± 0.5**

**Table 4:** Characteristics of genomes of *Pedicoccus acidilactici* ST6 and *Lactiplantibacillus paraplantarum* BPF2 strains.

LAB Strain	Protein-Coding DNA Sequences (CDS)	tRNA Genes	rRNA Operons Predicted
*P. acidilactici* ST6	1892	55	2
*Lpb. paraplantarum* BPF2	3326	73	3

**Table 5:** Gene ontology annotations results for each category.

Genome	Total Assigned GO Terms	GO Terms Molecular Function	GO Terms Biological Processes	GO Terms Cellular Components
*P. acidilactici* ST6	3683 GO terms in 1290 CDS (75%)	2063 (56%)	1199 (32.6%)	421 (11.4%)
*Lpb. paraplantarum* BPF2	5108 GO terms in 1909 CDS (67%)	2890 (56.9%)	1647 (32.2%)	553 (10.8%)

**Table 6:** Antimicrobial activity stability of bacteriocins from supernatant cultures of *Lpb. paraplantarum* BPF2 and *P. acidilactici* ST6 under different conditions of temperature, pH and treatment with enzymes.

Leucocin K from *Lpb. paraplantarum* BPF2		Pediocin-PA from *P. acidilactici ST6*	
Treatments	Bacteriocin Activity ^a^	Treatments	Bacteriocin Activity ^a^
Control (cell-free filtered)	24 ± 0.2	Control (cell-free filtered)	24 ± 0.0
**Heat**		**Heat**	
60 °C for 30 min	24 ± 0.2	60 °C for 30 min	24 ± 1
80 °C for 30 min	23 ± 0.5	80 °C for 30 min	24 ± 0.5
100 °C for 30 min	19 ± 1	100 °C for 30 min	17 ± 0.5
121 °C for 15 min	**-**	121 °C for 15 min	-
**pH**		**pH**	
2	24 ± 0.5	2	24 ± 0.5
3	24 ± 1	3	24 ± 1
5	24 ± 1	5	24 ± 1
7	24 ± 0.5	7	24 ± 0.5
9	24 ± 0.2	9	24 ± 0.5
10	24 ± 0.5	10	23 ± 0.2
11	-	11	**-**
**Enzymes**		**Enzymes**	
Trypsin	**-**	Trypsin	**-**
Papain	**-**	Papain	**-**
Proteinase K	**-**	Proteinase K	**-**

^a^ Diameter of inhibition zone in mm against *L. monocytogenes* DSM 112142.

## Data Availability

Bioproject PRJNA97852 included assembled genomes of *Lactiplantibacillus paraplantarum* str. BPF2 and *Pediococcus acidilactici* str. ST6 have been deposited at NCBI and are available under accession numbers GCA_030262435.1 and GCA_030262485.1 respectively.
